# Enhancing Methods for Research on Cannabis: A Workshop Report

**DOI:** 10.1186/s42238-025-00314-7

**Published:** 2025-09-29

**Authors:** Jonathan M. Samet, Jessica Barrington-Trimis, Lisa Bero, Ashley Brooks-Russell, Meghan Buran, Julia Dilley, Darin Erickson, Marilyn Huestis, Kent Hutchison, Thomas L. Jeanne, Michael Kosnett, David J. Kroll, Stephen Lankenau, Richard Miech, Rosalie Liccardo Pacula, Paula Riggs, Neeloofar Soleimanpour, Steven Teutsch, Gregory Tung, George Sam Wang

**Affiliations:** 1 Colorado School of Public Health, 13001 E. 17th Place, Aurora, CO 80045 USA; 2 University of Southern California, Los Angeles, USA; 3 University of Colorado School of Medicine, Anschutz Medical Campus, Aurora, USA; 4 Oregon Health Authority, Salem, USA; 5 University of Minnesota, Minneapolis, USA; 6 Thomas Jefferson University, Philadelphia, USA; 7 University of Colorado School of Pharmacy, Anschutz Medical Campus, Aurora, USA; 8 Drexel University, Philadelphia, USA; 9 University of Michigan, Ann Arbor, USA

**Keywords:** High-concentration cannabis, Delta-9-tetrahydrocannabinol, Public health, Exposure, Dose, Clinical research, Epidemiological research, Policy, Surveillance

## Abstract

**Aims:**

Progressive legalization of medical and recreational cannabis markets at the state-level has led to rapid growth of medical and recreational cannabis markets and to product diversification with emerging products having high concentrations of delta-9-tetrahydrocannabinol. Research on these products is still limited and the evidence available for policy formulation is diminished by methodological limitations.

**Methods:**

As a step towards addressing these limitations, the Colorado School of Public Health convened a multidisciplinary workshop that addressed four areas of cannabis research: epidemiological, clinical, surveillance, and policy. Workshop participants provided recommendations in each area to advance research on cannabis to make it more informative for decision-making on key policy topics. Emphasis was placed on assessment of use of cannabis products by study participants.

**Results:**

Recommendations for research methods and their implementation were made in the four areas. Those for epidemiology include using a core set of exposure assessment measures across three domains; developing this core set through a national and/or international scientific consensus process; ensuring the core set of measures are validated and readily available; and updating the core set periodically to account for ongoing changes in the cannabis landscape. Recommendations in the clinical research area include standard dosing and dosing terminology; standardized data collection instruments; identifying biomarkers for detecting cannabis exposure; and biological matrices. Policy research recommendations were offered for state regulators, evaluators/researchers, and policy makers. Surveillance recommendations include developing and implementing a novel and nimble surveillance system to monitor use of high-concentration forms of cannabis; adding questions to existing surveillance systems with the objective of monitoring high-concentration cannabis and adverse outcomes; and elevating the coordination, synthesis, and dissemination of findings in existing data sources that could signal adverse outcomes from high-concentration cannabis.

**Conclusions:**

Given the changing marketplace, it is urgent to improve the informativeness of cannabis research through enhanced research methods.

**Supplementary Information:**

The online version contains supplementary material available at 10.1186/s42238-025-00314-7.

## Introduction

The progressive legalization of medical and recreational cannabis has shaped the cannabis marketplace, bringing increasing concentrations of delta-9-tetrahydrocannbinol (THC) in cannabis products. A scoping review carried out by the Colorado School of Public Health found insufficient literature relevant to these contemporary products. (Bero et al. 2023) Additionally, the available evidence was limited by pervasive methodological problems, particularly obtaining a comprehensive and accurate assessment of products used. (Li et al. 2024) Rising use of hemp-derived psychotropic cannabis products, particularly hemp-dervived tetrahydrocannabinol compounds further complicates research on THC and health. (Harlow et al. 2022).

As a step towards addressing these problems, the Colorado School of Public Health convened a multidisciplinary workshop that addressed four areas of cannabis data gathering and research and evaluation: epidemiological, clinical, policy, and surveillance (See Supplemental Material for background). An overarching goal was to develop recommendations that would advance research on cannabis to guide decision-making on key issues, such as a regulatory limit on THC concentration. The participants (Supplemental Table 1) were charged with developing recommendations for characterizing use of cannabis products that would lead to more standardized assessments and that would support harmonization of findings across studies and coalescing evidence from surveillance systems and research.

**Fig. 1:**
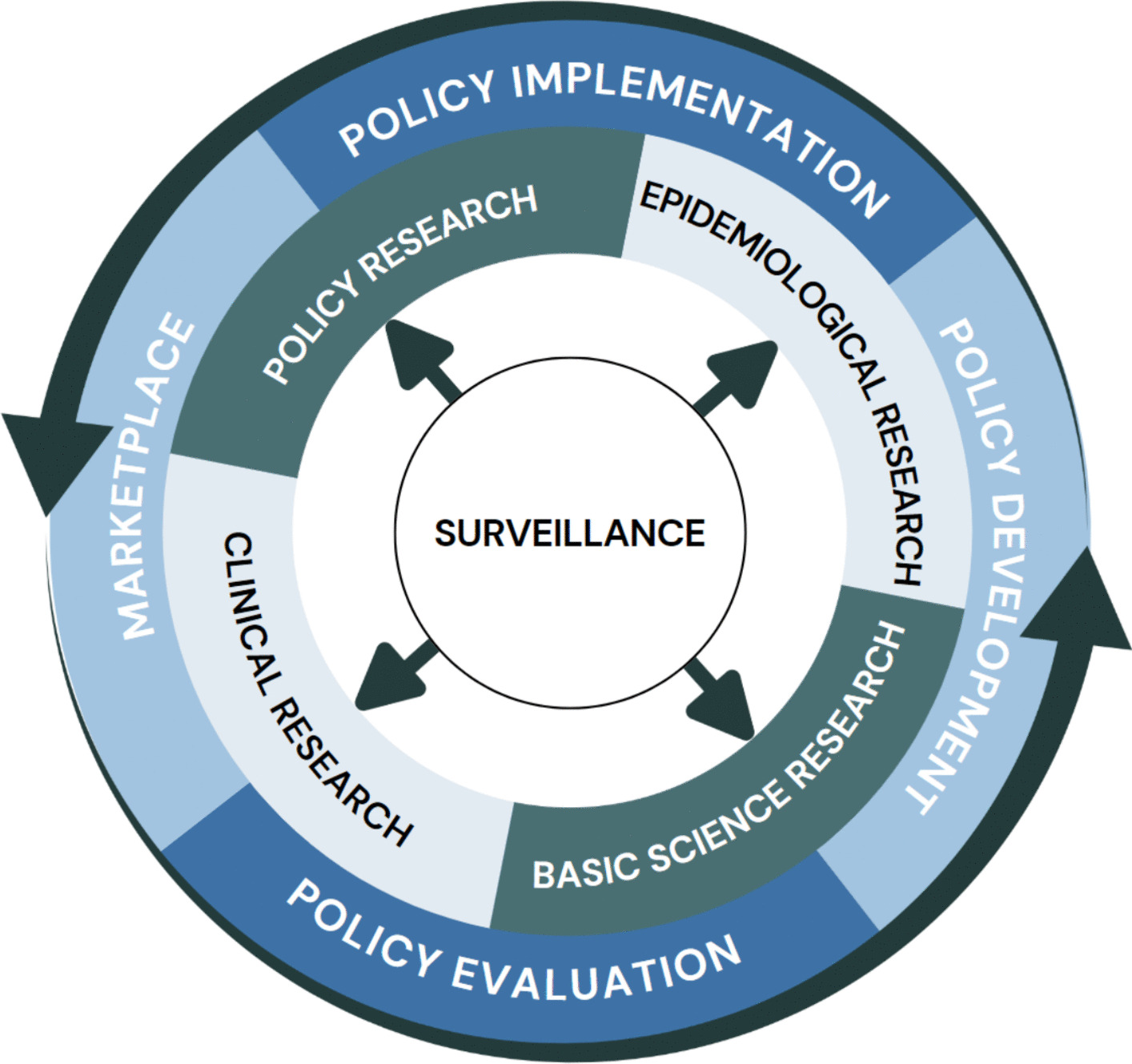
The evidence-based policy cycle to improve cannabis policy for communities

To illustrate the interlocking of these four areas, workshop participants adapted a general framework (Fig. 1) depicting the centrality of public health data and how the “real world” provides the questions that drive research. Policy occupies the outer ring in a loop that relates to research and surveillance and that is dynamic, changing with findings of research, signals from surveillance, and policy evaluation. Recommendations in the four areas are accompanied by a rationale and comments concerning implementation.

We note that the workshop was held in June 2024 in advance of the release of the National Academies report, “Public Health Consequences of Changes in the Cannabis Policy Landscape.” (2024) That report offered an agenda for research on cannabis policy and called for research on the health effects of emerging cannabis products, stressing the need to address “emerging synthetic and semisynthetic cannabinoids and high-concentration products.” (2024) The National Academies report did not address research methodology, the focus of the June 2024 workshop convened by the Colorado School of Public Health. (2024).

## Epidemiological Research

### Improving Exposure Assessment for Observational Studies

**Fig. 2:**
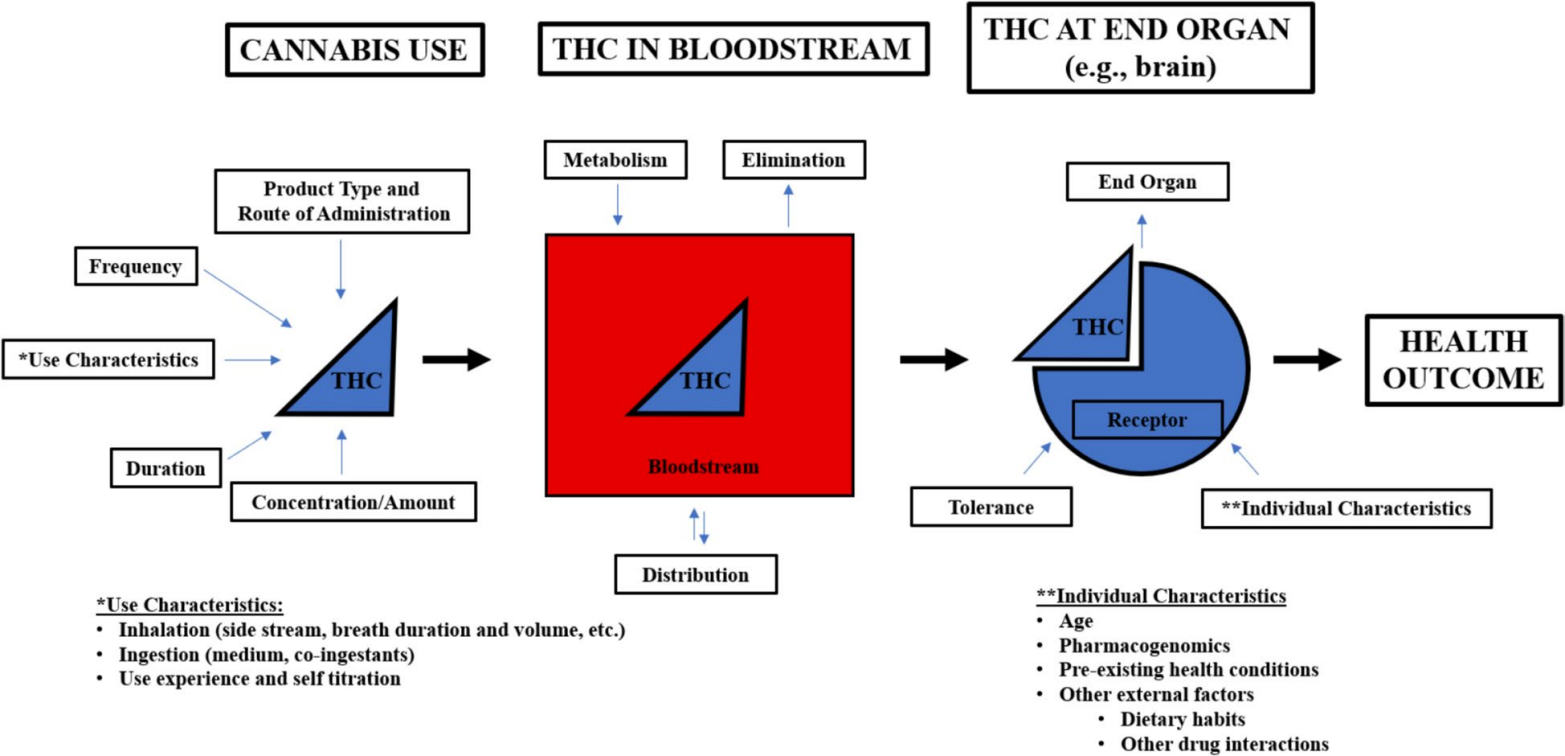
Pathway from cannabis use to health outcomes

Epidemiological studies and public health surveillance provide data on use of cannabis products in “real world contexts.” Epidemiology addresses questions about determinants, consequences, and natural history of use while surveillance characterizes who is using cannabis products and how they are being used. Cannabis use is complex, with dimensions including amount, form, mode of administration, frequency, and duration (Fig. 2), leading to challenges in understanding the products that people are using, how they are using these products, and the situations and the factors that contribute to use.

Due to resource and practical limitations, as well as the emphasis on measuring real-world use, many observational studies solicit self-reports of cannabis products used. (Li et al. 2024) Exposure assessment strategies in observational studies assume that people can accurately remember and report their use behaviors over specified periods of time. Such information is the basis for inferring THC exposure. Determining levels of ‘exposure’ to cannabis use is particularly challenging given the changing cannabis landscape and evolution of the cannabis products available in the market, inaccurate labeling of the amount of THC contained in them (2023), and how they are used. There have been efforts to develop standardized measures to facilitate work done internationally. For example, Lorenzetti and colleagues (Lorenzetti et al. 2022a) offer consensus recommendations on a minimum set of indicators to measure cannabis use, but these have not been widely adopted and are not specific for high concentration products. The recommendations were focused on assessing behaviors associated with the number of times a product was used and the weight of cannabis used, not the amount of THC.

One major methodological problem noted in the scoping review of high-concentration cannabis products (2023; 2024) was highly variable exposure assessment methods such that data from multiple studies could not be combined in any useful way. To address this problem, we propose a comprehensive list of constructs to be evaluated in epidemiologic studies—including a standardized core set of items—that can be used to characterize exposure to THC (Table 1).

**Table 1:** List of Potential Exposure Assessment Items

**Product Characteristics (regulatable, potentially)**	Type of product(s) used (primary: flower, concentrate, edible) Intended route(s) of administration (inhaled, ingested, dermal, etc.) Concentration of cannabinoids Ratio of CBD to THC Active ingredients (THC, CBD, hemp-derived psychoactive, etc.) Terpenes/secondary ingredients Serving size and doses/package Packaging (labeling, health claims, warnings, etc.) Testing for trace contaminants, mold and other potentially unhealthy contents
**Use Behaviors**	Frequency of use of specific products (number of days per month, sessions per day, puffs per session) Duration (how long have they been using regularly) Duration (how many minutes/hours is a typical use session)? Actual route of administration Age of initiation Polysubstance use—both poly-cannabis use and use of cannabis + nicotine use (Self-titration to desired effect) Intoxication level Tolerance/escalation of use Unintentional use (e.g., ingestion by babies)
**Contextual Factors**	Setting User group Reason for use Desired effects of use Access to/availability of product (source) State/jurisdictional factors Sociocultural Marketing

### Development of Core Exposure Assessment Items

To assure widespread acceptance of a standardized approach to exposure assessment, a consensus process should engage key professional organizations, funders, and members of the research community. In advance, a systematic review of exposure survey questions is needed to identify and group exposure assessment measures relevant to product characteristics, behavioral patterns of use, and contextual factors. In addition, the creation of a database that lists questions used in major published cannabis studies would be a useful resource for the consensus committee. For example, questions from established surveys at international, national and state levels could be compiled (Table 2). Workshop participants developed a preliminary list of measures to be included under each construct (Table 1). Table 3 shows example questions that fall under the category of cannabis use.

**Table 2:** Key International, National, and Statewide Surveys Described

Survey Reach	Survey Name and Responsible Entity	Survey Description	Timing of Data Collection
International	International Cannabis Policy Study (ICPS) (2024)	ICPS is a survey that uses a quasi-experimental research design that examines the different cannabis laws and the impact of the legalization of cannabis in six countries: Canada and the United States since 2018, Australia and New Zealand since 2021, and the United Kingdom and Germany since 2023. Survey respondents are between 16–65 years old and new respondents participate annually. The surveys are revised each year to capture trends and policies, and they are adapted for each country. More than 300,000 respondents have completed the survey	Collected Annually and Last Reported on US data in 2022
	International Cannabis Toolkit (iCannToolkit) ( Lorenzetti et al. 2022a)	Tri-layer framework was developed to harmonize evidence on cannabis consumption, harms, and mitigation approaches. This toolkit was developed by 25 international multidisciplinary cannabis researchers in 2021. This framework specially asks questions focused on if a respondent has ever used cannabis, the last time they used cannabis, how many times they used cannabis in the last 30 days, setting specific questions, including history of use, frequency of use, and amount of cannabis use, and various ways to gather biologic measures when using cannabis. Data using this framework is not available publicly yet	Data are not publicly available at this time
National	Behavioral Risk Factor Surveillance System (BRFSS) (2022) *Centers for Disease Control*	National telephone survey collecting state-level data regarding U.S. residents on topics focused on health-related risk behaviors, chronic health conditions, and use of preventive services. This survey was established in 1984 with 15 states but has expanded to now gather data on all 50 states, the District of Columbia, and three U.S. territories. Data are reported annually with more than 400,000 adults interviewed each year. This is the largest continuous health survey system in the world	Collected Annually and Last Reported in 2023
	Monitoring the Future (MTF) (2024) *University of Michigan*	Evaluating the attitudes of young people starting in 8th to 12th grade to monitor trends in substance use and abuse. The MTF study, carried out by the University of Michigan, was initially sent out to high school seniors in 1975 and extended to 8th graders and 10th graders as of 1991. Over 100 different schools and 10,000 respondents across the United States in each grade are surveyed. MTF has surveyed over 1.7 million students – initially starting with mail-in surveys, but MTF has now transitioned to a web survey. Up to 350 students from a single school may be included. Starting with the class of 1976, 2,400 randomly selected respondents were sampled to complete a follow-up survey every two years from ages 19–30 and every five years starting at age 35	Collected Annually and Last Reported in 2024 Report
	National Drug Early Warning System (NDEWS) (2024)	Examines trends on substance use starting in 2014 across the United States. This was a NIDA funded initiative to provide real-time surveillance to identify early signals of potential drug epidemics. Data are updated and reported continuously from 16 sentinel sites across the United States	Dashboard Weekly Briefings
	National Poison Data System (NPDS) of America’s Poisons Centers (2024)	Report of poisoning data surveillance system for the United States across 55 poison centers. The NPDS produces an annual report each September. NPDS was launched on April 12, 2006, and previously data was collected through the Toxic Exposure Surveillance System (TESS) in 1983 and close to real-time data as of 2003. Data are reported via phone calls to the National Poison Help Line, poison centers, and the website. Data are collected on a variety of elements like age of patient, animal type, chronicity of the condition, clinical effect and duration, exposure site and duration, level of health care facility, state, start date, route of exposure, reason for exposure, substance quantity/formulation/certainty, treatment therapy, weight of patient, year when incident occurred, and others	2022 Data 2022 Annual Report
	National Survey on Drug Use and Health (NSDUH)(54)	Collects data on substance use disorders, tobacco, alcohol, and drugs, receipt of substance use and mental health treatment, and mental illness at a national, state, and substate level. The Federal Government started conducting this survey in 1971. In 1999, the survey encompassed all 50 states and the District of Columbia and shifted from paper and pencil data to computer-assisted interviewing by NSDUH staff. As of 2002, NSDUH provides a $30 incentive for respondents and in 2020 they transitioned to web-based interviewing while maintaining their in-person home interviews. NSDUH reports on individuals ages 12 and older in the United States and the District of Columbia. Individuals who are experiencing homelessness and are not utilizing a shelter, active military personnel, and residents of institutional group residents are excluded from the survey	Collected Annually and Last Reported in 2023
	Pregnancy Risk Assessment Monitoring System (PRAMS) (2024)	Identifying groups of women and infants at high risk to learn why some infants are born healthy and others are not. PRAMS was developed in 1987 to reduce infant morbidity and mortality using data relating to maternal behavior before, during, and immediately after the live birth. Respondents across the United States who have recently given birth are picked randomly from the birth certificate registry. Surveys are distributed by mail between state, territorial, or local health departments and the CDC’s Division of Reproductive Health. If there is no response from repeat mailing, then respondents are contacted via a telephone interview. Starting in 2023, responses can be submitted online. Each participating site has 1,000–3,000 sampled respondents annually	Collected Annually and Last Reported in 2022
	ToxIC Core Registry of the American College of Medical Toxicology (2024)	Patients evaluated and treated by a medical toxicologist experiencing effects from exposure to medications, illicit drugs, metals, pesticides, natural substances, and other chemical substances. The registry was established in 2010 and over 90,000 cases were reported by trained medical toxicology physicians across the United States. Annual reports are published in the Journal of Medical Toxicology to summarize the year’s cases	Collected Annually and Last Reported in 2022
	Youth Risk Behavior Surveillance System (YRBSS) (2021) *Centers for Disease Control & Prevention*	YRBSS was established in 1991, and they were conducted biennially to gather data on students in grades 9–12 in the United States at the national, state, and local levels. YRBSS gathers self-administered data that students record on a computer-scannable answer sheet on health-related behaviors and experiences that contribute to leading causes of death and illnesses during adolescence and adulthood. Students, schools, and districts are randomly selected. Four states (Minnesota, Oregon, Washington, and Wyoming) do not participate in the state surveys	2013–2023 Summary Report Collected Biennially (every odd year) and Last Reported in Data from 2021
State Level Colorado Specific	Baby and You (2024)	This tool was developed to further understand those who are postpartum in the state of Colorado. The survey program was created by HB 22–1289 and launched in spring 2023. About 3,400 postpartum people are selected each year from the birth certificate record to join an online platform to complete this survey 3–4 months postpartum. Surveys are sent at 3–4 months, 1 year, 2 years, and 3 years postpartum	Data not available yet
	Health eMoms (2024)	Examined the experiences of people who were postpartum in Colorado. This survey has now transitioned into Baby and You, Listed above. Previously this survey was carried out from 2018- 2021 where 2,400 postpartum people in Colorado were invited to join the online survey and complete 1–2 brief surveys. Participants received an electronic gift card for completion of each survey	Collected Annually and Last Reported in 2021
	Health​y​ Kids Colorado Survey (HKCS) (2024)	Evaluates the health and well-being of young people in Colorado in grades 6–12. Since 1991 the state of Colorado has surveyed young people about their health. In 2013, the survey was combined with other state surveys to enhance data collection efforts. The survey is administered in the fall of odd-numbered years to participating schools in Colorado and the state health department publishes statewide and regional results in the summer of even-numbered years. Participating schools receive a summary report of their results within 4 weeks of administration. In 2023, 239 schools participated in this survey	Collected Biennially (every odd year) and Last Reported in 2023
State Level Washington Specific	Daily Sessions, Frequency, Age of Onset, and Quantity of Cannabis Use Inventory (DFAQ-CU) ( Cuttler and Spradlin 2017)	This tool was created to analyze cannabis use over a multitude of factors in Washington. Between September 2015 and May 2016, more than 2,000 college-aged undergraduate students at Washington State University who were enrolled in eligible Psychology courses and met the remaining inclusion criteria were sampled. Students answered an online self-reporting to measure frequency, age of onset, and quantity of cannabis use	Last reported in 2016
	Washington Young Adult Health Survey (WYAHS) (2014)	The WYAHS is an online survey developed to analyze health behaviors in young adults that started in Spring 2014. Each year new Washington young adults (ages 18–25) are invited to complete the survey in addition to those who have completed the survey in the past to evaluate if and how behaviors may change over time. Return participants may be over the age of 25	Last reported in 2021

**Table 3:** Example questions regarding cannabis use

Questions	Answer Options
Ever used cannabis?	Yes No
Past year use	No Once or twice Monthly Weekly or more frequently
Past month use (30 days)	a. Yes b. No
Age at first use	Provide age at first use
On a typical day when you use:	How many sessions/day? (Alt. times used per day) How much use/session? (Alt. amount used per time of use) Smoke: Number of Puffs Vape: Number of Puffs Concentrate/Dab: Number of Hits/Inhalations Edible: Amount of THC in Milligrams Tincture: Number of Drops Do you use to a desired effect?
Which of the following types of products have you used (check all that apply)	Flower Do you know THC% of the flower you use? Do you choose flower based on THC%? Indica/sativa/hybrid Pre-roll Smoke/dry vape Water pipe Carts Do you know THC% of the carts you use? Do you choose carts based on THC%? Indica/sativa/hybrid Disposable/refillable Edibles Do you know mg THC in the edibles you use? Do you choose edibles based on mg THC Average milligram session of edibles Gummy/chocolate/beverage Indica/sativa/hybrid THC/CBD ratios

If the recommended consensus process is pursued, those engaged should establish a priori criteria for inclusion of clearly defined items at the start of the consensus process. Emphasis should be placed on core questions requisite for all health outcomes, acknowledging that more focused questions are needed for particular outcomes. For example, research directed at respiratory vs. mental health outcomes or chronic vs. acute outcomes may require differing and specific measures to be added in the core set in order to link exposure to outcome. Measures should be developed for all types of research and for surveillance.

To establish priorities, key barriers to using core sets of measures need to be identified and addressed. One barrier is the increasing complexity of products in the marketplace, making a complete assessment of all products used time-consuming, and possibly soon out-of-date. Additionally, assessment of dual use with nicotine-delivering products may be relevant for some groups, e.g., adolescents. (MacCallum et al. 2024) Participants gave priority to items with established validity and reliability, as well as items validated with biomarkers. One indication for giving priority is relevance to decision-making by policymakers, e.g., patterns of use of vape pens. For example, product characteristics such as THC concentration are more amenable to regulation than use characteristics. Lastly, “not recreating the wheel” should guide the selection of measures (and their specific wording) to avoid replicating items that are already used.

Also, use of a core outcome set, i.e., a standardized assessment, facilitates comparisons of results across studies and their combination for evidence synthesis. For example, the Core Outcome Measures in Effectiveness Trials (COMET) Initiative brings together people interested in the development and application of agreed standardized sets of outcomes. (2024).

### Recommendations for Epidemiological Research

1. ***Use a core set of exposure assessment measures for all epidemiological and surveillance studies****. Rationale*: Non-comparable approaches limit interpretation of the literature and of evidence synthesis and integration.
  Measures should address product characteristics, behavioral patterns of use (including co-use with alcohol, nicotine, and other drugs, age of initial product use, chronicity of exposure, amount of product used, frequency of product use), and socio-contextual factors pertaining to use. A core set of measures should be used and reported consistently across all studies and supplemented with additional items for individual studies as needed.
2. ***A team of experts should develop this core set through a national and/or international scientific consensus process****. Rationale*: A transparent and stakeholder engaged process will facilitate acceptance.
  A national and/or international group of cannabis researchers with broad expertise, funders, and individuals from affected groups (e.g., youth who use cannabis and individuals employed in the cannabis industry) should be convened to develop the core measures. Criteria for identifying items include their methodological rigor and utility for evaluating cannabis use in the population context.
3. ***The core set of measures should be validated and freely available.**** Rationale*: Validity should be established before use to assure data quality. Accessibility will promote wide adoption of core measures.
  Validation studies should be conducted to assess reliability and internal and external validity across different populations (with intentional focus on minoritized populations). Developed measures should be available online to download in text, REDCap, and Qualtrics formats for facile implementation in studies.
4. ***The core set should be updated periodically to account for ongoing changes in the cannabis landscape****. Rationale*: To remain current with the rapidly advancing marketplace, changes in products and context will require periodic updating.
  The core set of measures should be reassessed on a regular basis to ensure that new products (or characteristics of products), behavioral patterns of use, and contextual factors surrounding use are incorporated. The need for such updates should be based on findings of surveillance of cannabis products and their use.

### Implementation of Recommendations for Epidemiological Research

Research funders and researchers should take responsibility for diffusion and systematic use of the core exposure assessment measures. Since research depends on funding, funders could support development of the measures by supporting consensus meetings and validation studies. The funders could request that cannabis researchers use these measures in research. There would be beneficial harmonization if a common core set of measures were implemented in epidemiological studies, surveillance surveys, and prospectively planned research syntheses. Journal editors could implement reporting standards to foster adoption of core exposure assessment measures.

### Clinical Research

Clinical research on cannabis includes studies evaluating the safety and therapeutic effectiveness of cannabis and its components (cannabinoids) on human health, such as clinical trials on outcomes considered beneficial, e.g., moderating the side effects of chemotherapy, pharmacokinetic and dynamic studies, and observational studies in clinical populations. (Ward et al. 2021; Huestis 2007; Solmi et al. 2023; Bialas et al. 2022) Cannabis research should follow established guidelines, such as the Consolidated Standards of Reporting Trials (CONSORT) (2024) statement for clinical trials, and basic research principles. (Kandi and Vadakedath 2023) In addition to these standards, we propose additional recommendations to improve the rigor and accuracy of findings specific to cannabis use.

### Recommendations for Clinical Research

1. ***Dosing and dosing terminology should be standardized***. *Rationale*: Incomplete product descriptions and varied terminology complicate interpretation of the clinical research literature. Without standard terminology, cannabinoid dose cannot be accurately measured.
  The current marketplace has diverse products (Table 4). THC amount varies in these products, with flower ranging between 10 to 30%, and concentrate products between 50–90%. (2020) Without standards for reporting dose, it is difficult to determine or estimate an individual’s actual intake of cannabis (Fig. 2). The workshop participants propose to use the term “available dose” as the amount of the cannabinoid of interest within a product, recognizing that intoxicating THC can be derived from hemp-products and thus should be considered part of the standards for describing THC content more generally. This quantity should reflect the current cannabis marketplace standards for THC content, which include amount (mg) and percentage by weight of total THC cannabinoids (delta-9 tetrahydrocannabinol + tetrahydrocannabinolic acid) in inhalational products and milligrams (mg) of cannabinoid (serving size and total in an item or package) for ingested products. (Cash et al. 2020; MacCallum et al. 2023) To the extent possible, clinical trials should standardize dosing of THC and cannabinoids, including the cannabis product used. Additionally, there should be standards and permission for cannabis product testing for cannabinoid content in order to ensure accuracy of available dose. Current federal restrictions make handling and testing products difficult for researchers. Details on use characteristics should be standardized. Products sold to the public should be available for research.

**Table 4:** Cannabis products available on the marketplace and typical THC amounts by product as summarized by workshop participants

	**Type of Product**	**How Consumed**	**Amount (THC) in the Products**
Plant	Flower	**Product Type:** Flower **Mode:** Joint, bowl, bong, pipe, bong **Onset:** Within minutes **Duration:** Lasts about 1—3 h	**Typical Range: 10—25% THC** 1 gm Joint = 100—250 mg THC
Concentrates	Hash, budder, wax, sugar, shatter, resin, oil, and 500 mg cartridges for example	**Product Type:** Concentrates **Mode:** Vape pen and dab rig **Onset:** Within minutes **Duration:** Lasts about 1—3 h	**Typical Range: 50—95% THC** Vape 1 mL Cartridge = 500—1000 mg THC 1 Puff = 3—5 mg of Oil or Concentrate 500 mg Cartridge = 130—140 Puffs
Edibles	Baked goods, candies, powders, and beverages for example	**Product Type:** Edibles & Drinks **Mode:** Ingestion **Onset:** Within 30 min—2 h **Duration:** Lasts about 1—8 h	**Typical Range per Serving Size: 5—10 mg THC**
  The “absorbed dose” is the amount of cannabinoid that is absorbed and distributed into the systemic circulation. This is an estimated parameter that may vary with route of use, partly due to bioavailability. Pharmacokinetic studies show that gravimetric assessments of THC available dose, such as estimating the mass of THC in a cannabis product that is combusted or volatilized, may be an imprecise measure of absorbed dose due to variable inter-subject smoking topography, and differences in efficiency of drug extraction associated with cannabis use history. (Henthorn et al. 2024) There is considerable inter-subject variability in pharmacokinetics of THC and other cannabinoids. (Lile et al. 2013; Newmeyer et al. 2016; Vandrey et al. 2017; Rozanc et al. 2024; Berl et al. 2022) Pharmacogenomics also play a role in cannabinoid pharmacokinetics and pharmacodynamics. (Hryhorowicz et al. 2018) We note that some evidence indicates that the THC:CBD ratio also affects THC bioavailability and occurrence of psychological effects. (Freeman et al. 2019).
  Finally, the label “high-concentration” should be restricted to products with higher THC amounts than found naturally in plants. These products are created by extracting THC from the plant using various techniques and solvents and concentrating it. The term “high-potency” should not be used to describe a high-concentration THC product, as pharmacologic potency of THC is not dependent on concentration.
2. ***Data collection instruments used in clinical research studies should be standardized****. Rationale*: To facilitate comparisons across studies, standardized characterizations of research participants, their cannabis use patterns, and key outcomes should be implemented. Lack of standardized data collection instruments limits comparisons among studies and the generalizability of findings.
  Instruments for data collection should be standardized and validated, if possible, for describing patterns and characteristics of cannabis use. Variables to be standardized include route, frequency, chronicity, and type of product. Information gathered on frequency of use can be standardized using proposed inventory measures such as the International Cannabis Toolkit (iCannToolkit) or Daily Sessions, Frequency, Age of Onset, and Quantity of Cannabis Use Inventory (DFAQ-CU). (Lorenzetti et al. 2022a; Lorenzetti et al. 2022b; Cuttler and Spradlin 2017; Gette et al. 2023) Detailed data are collected using 30-day timeline follow-back surveys.
3. ***Biomarker development is needed for detecting cannabis exposure and characterizing absorbed dose.**** Rationale*: Availability of and consistency in using accurate biomarkers would facilitate research and surveillance. The use of validated biomarkers will give insights into the accuracy of questionnaire assessments of use of cannabis products.
  Biomarkers used to detect and quantify absorbed dose need to reflect the complexities of cannabinoid pharmacokinetics. THC is metabolized in the Liver to the active metabolite 11-OH-THC and further to an inactive metabolite 11-nor-9-carboxy-THC (THC-COOH). Glucuronidation of all three analytes is extensive. (Huestis 2007; Lucas et al. 2018) After inhalational use, THC quickly (within seconds) appears in the bloodstream and partitions into the brain. (Huestis 2007; Lucas et al. 2018) After ingestion, THC peaks at a lower blood concentration than with inhalation and reaches a peak within several hours. (Huestis 2007; Lucas et al. 2018) 11-OH-THC/THC ratios are higher after oral ingestion. THC-COOH peaks later and has a slower decline. (Vandrey et al. 2017; Nadulski et al. 2005) Additionally, THC-COOH can accumulate in the blood with chronic cannabis use (Kosnett et al. 2023; Karschner et al. 2009), and the concentration of THC-COOH in blood reflects cumulative THC exposure over the past several weeks to months. (Fabritius et al. 2014) The carboxy metabolite is the primary form of THC excreted in urine. Most THC and other cannabinoids are excreted in the feces. (Huestis 2007; Lucas et al. 2018) Thus, timing for measurements of THC and metabolites in the bloodstream is dependent on route of exposure and what outcome measurements are evaluated. Another consideration is whether THC and metabolites are measured in whole blood or plasma. Whole blood concentrations of THC and metabolites are approximately half plasma concentrations due to high plasma protein binding and limited distribution into erythrocytes. (Schwilke et al. 2009).
  The biological matrix selected for analysis is also key. (Hubbard et al. 2021) THC and metabolites are present in multiple biological matrices: blood, urine, oral fluid, feces, hair, sweat, and breath. Determining which matrix to use depends on the outcome measures of interest and feasibility considerations, such as timing of last use in relation to sample collection. Route of consumption and frequency of use influence the optimal time for measuring the amount of THC absorbed. Blood concentrations of cannabinoids are essential for pharmacokinetic and pharmacodynamic studies and may offer information on the extent and temporal pattern of cannabis use. Measurements in oral secretions are increasingly used in clinical studies for detecting recent cannabis use, and methods for breath cannabinoid measurement are being developed. (Vandrey et al. 2017; Lee and Huestis 2014; Robertson et al. 2022; DeGregorio et al. 2021). If validated, they could be used for screening in the workplace and for assessing cannabis use and driving impairment. THC-COOH in urine reflects prior cannabis use and may not identify the presence of acute pharmacodynamic effects due to the prolonged elimination of THC metabolites in naïve and chronic users. (Connors et al. 2020) Oral fluid, hair, and urine (normalized to creatinine) levels can be helpful for monitoring abstinence and longer-term use. The perinatal population is unique with matrices for both maternal and fetal exposure. For fetal exposure, measurement in meconium can detect maternal use in the last trimester and in the umbilical cord for the last few weeks of pregnancy. (ElSohly and Feng 1998; Pandya et al. 2023; Jensen et al. 2019; Kim et al. 2018; Metz et al. 2022).

### Implementation of Recommendations for Clinical Research

We recommend the standardization of several elements for clinical studies on cannabis: terminology, data collection, and biomarkers. Such standardization should be encouraged by funding agencies at multiple levels including state funding mechanisms that might support surveillance or research, the private sector, and national research funders, particularly the National Institutes of Health (NIH). Standardizing clinical studies on cannabis will assist in collaborative efforts between research consortiums and organizations, allowing for the synthesis of data and improving our understanding of the health impacts of cannabis.

### Policy Research

United States (US) cannabis policy is shaped by decisions made at national, state, county, city, and even neighborhood levels (with zoning decisions, for example). (2024) Interactions across levels are also relevant, particularly with the current situation of national prohibition and legalization by some states. Discussion in the workshop focused on state-level policy for two reasons: (Bero et al. 2023) in the US, the states are the primary venue for policy activities, including cannabis legalization; and (Li et al. 2024) measurement of the degree to which local policies reinforce or conflict with a given state policy can be captured through careful policy and legal research. The key actors involved in state policy include: 1) *State regulators* with a critical role in both development as well as implementation and enforcement of cannabis policies. Regulators are also key players in data access, data collection, and surveillance, all components of the policy cycle. 2) *Policy Makers* with a critical role in developing new policies and engagement with other key players in the policy cycle in using findings from research and evaluation. 3) *Evaluators and researchers* whose role in the policy cycle is to engage in policy evaluation, surveillance, and research that feeds back to state regulators and policymakers. The recommendations below are directed towards these groups.

### Recommendations for Policy Research

1. ***For State Regulators — Develop and implement methods for assessing the effectiveness of implementation and enforcement of existing cannabis rules and regulations, including civil penalties and reporting****. Rationale*: At present, methods for such assessments are ad hoc and do not provide optimum information for decision-making.
  Effective implementation and enforcement of existing cannabis policies will ensure that state cannabis marketplaces are, in fact, governed by the rules and regulations in state legislative and administrative law. Variations in state-to-state cannabis policies provide a natural experiment for researchers to learn about effectiveness. The findings of policy evaluation research are needed to understand what policies at the state-level are effective at achieving their objectives and mitigating harms from cannabis use.
2. ***For State Regulators — Make existing data regarding the cannabis market and population level health outcomes available for research and analysis, feeding results back to policy makers.****Rationale*: For policy formulation and evaluation, the full range of data on cannabis sales, products, quality, and utilization is needed without restriction.
  Effective surveillance is critical to understanding what impact policies are having on the cannabis marketplace and how that is available in the marketplace is impacting public health. The data needed include elements of the cannabis market, such as numbers detailing seed to sale quantities (by product unit), and testing data on products sold. Effective surveillance also requires health related data to monitor the population-level health impact. Examples of needed market and health data include:
  *Behavioral Health Surveys.* National health surveys, such as the Behavioral Risk Factor Surveillance System (BRFSS)(2022) and the Youth Risk Behavior Surveillance System (YRBSS)(2021), as well as state-specific surveys such as the Healthy Kids Colorado Survey (HKCS)(2024), play a critical role in monitoring trends in youth cannabis use, products consumed, and exposure to marketing (Table 2). These data need to be paired with valid exposure data to more clearly identify the health implications from cannabis use.
  *Health System Data.* Health systems data include electronic medical records, claims, and prescriptions. These data sources are critical for monitoring and measuring more detailed healthcare utilization and health outcomes associated with cannabis products available in the marketplace. These data need to be paired with valid exposure data to more clearly identify the health implications of cannabis.
  *Market Data Linked to Census Tract, Neighborhoods, and Schools.* Market data in the form of sales by product are critical to understanding how much and what types of cannabis products a given neighborhood or population is exposed to. Having these data as granular as possible would provide valuable population-level exposure data to bridge current gaps regarding self-reported use and health outcomes.
  *Patient and Consumer Purchase Panel Data.* Specific purchase data from patients or consumers, would provide further detail on cannabis use patterns. The pairing of detailed biomarker and outcomes data with patient and consumer purchase data would support analyses addressing the health implications for specific product use patterns. For example, the availability of such data would facilitate research regarding the extent to which consumption of high-concentration cannabis products may be associated with extensive, chronic cannabis use and exposure to high-concentration THC doses.
3. ***For State Regulators — Initiate the collection of new data needed for effective surveillance of state-level cannabis markets and health outcomes.**** Rationale*: Existing data systems should be complemented by tracking products closely and identifying sentinel events.
  Here, we propose collection of data not currently available on a routine basis. Examples include the following:
  *A Product Registry System.* Implement a comprehensive product registry system with information such as the name/location of the farm, harvest date, pesticides used, additives used, testing results, product concentration, and processing methods. This type of product registry, which exists in Massachusetts, can more specifically link health outcomes to detailed product characteristics not captured in current market data.(2024)
  *Adverse Events Tracking System.* Improving the current formal reporting process of adverse events to capture greater detail on negative health effects of specific cannabis products used, accompanied with a product registry could effectively identify negative health impacts associated with specific cannabis products. Formalized reporting of cannabis related events from hospital emergency department records, postmortem exams, citations for driving under the influence (DUI), and suicides could be included and further aid in the identification of negative health effects from specific product characteristics. Medical toxicology data such as that compiled in the ToxIC Core Registry of the American College of Medical Toxicology ((American 2024)) and the National Poison Data System of America’s Poisons Centers (2024) may offer detailed information on the association of specific cannabis products with acute adverse health events in adults and children.
4. ***For Policy Makers – Communicate needs to researchers and academics and create the structures to facilitate access to the data and research needed to inform effective policy development and Evaluators/Researchers — Engage in “directed” research at the request of policy makers and state regulators. What do policy makers need and want?**** Rationale*: Researchers and policy makers should be connected and communicate so that critical evidence gaps can be filled on an as-needed basis.
  This recommendation is focused on creating interfaces between researchers and policy makers to ensure that (1) researchers are conducting policy-relevant research and evaluation; and (2) policy makers are making the data needed for surveillance and research available. This recommendation is also focused on facilitating effective communication among the key actors in the policy cycle to more effectively surveil, identify specific public health concerns, and develop and implement policies to address those public health concerns.
  Evaluators and researchers should partner with policy makers to provide evidence that aligns with the policy needs of states. In addition, researchers should adhere to the state-of-practice in translating evidence to policy makers and to make the policy implications of their results explicit and clear as possible.
5. ***For Policy Makers – Develop new and innovative policies to address harms of cannabis use based on the best available evidence.**** Rationale*: In the policy cycle framework, the elaboration of evidence-informed policy is the objective.
  The growth and development of cannabis marketplaces have moved more quickly than the scientific research needed to understand the public health implications of those marketplaces and products. Policy development and experimentation based on the best available evidence is appropriate, especially when paired with rigorous evaluation to assess the impact of new policies. Policy areas that show promise to combat the harms from cannabis use include implementing (Bero et al. 2023) taxes on products, (Li et al. 2024) age restrictions, and (Harlow et al. 2022) product registration and/or restrictions. (2022; 2020).

### Implementation of Recommendations for Policy Research

Implementation of these recommendations requires coordination and collaboration among the key actors in the policy cycle (Fig. 1). We organized and presented our specific recommendations by the key actors we see as having the most direct control over the implementation of the recommendation. However, each recommendation feeds into the broader policy cycle and only through coordination and collaboration between all the key actors, can the policy cycle respond rationally and more effectively to address the public health impact of cannabis legalization and state marketplaces.

### Public Health Surveillance

Public health surveillance activities include monitoring trends over time to help identify emerging issues and shifts in behaviors, identifying populations or communities that are particularly vulnerable, enabling more targeted interventions and resource allocation, and tracking the impact of policy measures. Surveillance data can help allocate resources more effectively to ensure that areas with greatest need receive appropriate support and funding, and to inform future directions for research and prevention programming.

Population-based surveys are critical to surveillance. For adults, the BRFSS (2022) and the National Survey on Drug Use and Health (NSDUH)(Substance 2024) are most relevant. For adolescents these include the NSDUH, Monitoring the Future, and the YRBSS (2021). The BRFSS and YRBSS have variations in state implementations. The International Cannabis Policy Study (ICPS) (Hammond 2024) is emerging as a cross-national resource. The Pregnancy Risk Assessment Monitoring System (PRAMS) covers the prenatal period (2024) (Table 2). In addition to these national or nationally coordinated systems, there may be state-specific surveys, which have greater flexibility and responsiveness to track the use as the marketplace changes.

Population-based surveys are not the only source of public health surveillance data. Other potentially useful data, some collected for administrative purposes that could be more fully leveraged for public health surveillance, include:

- Motor vehicle related data, including crash data, injury and fatality data (2024), forensic testing results, court records for charges, arrests and convictions.
- Market data, such as dispensary licensing, sales and tax data, and product testing results.
- School and educational system data, such as absenteeism and discipline related to drugs, and surveys of schools on policy and prevention programming e.g., School Health Profiles. (Fadus, et al., 2021) To be most informative, such data should include necessary demographic data to allow analysis by age, race, ethnicity, and other important subgroups. (CDC 2022) When available, such information will also enhance the utility of data related to adults.
- Hospitalization and emergency room visits related to cannabis use.
- Poison Center (2024) and ToxIC Core Registry of the American College of Medical Toxicology (2024) reports related to cannabis use.
- Vital records data.
- Adverse event reporting data provided to the Food and Drug Administration (FDA) (2023).

The emerging issue of high-concentration cannabis use should be given priority in surveillance systems. High-concentration products are nearly synonymous with extracts or manufactured products, and in this way are a particular concern for a growing number of states with legal use by adults or medical use programs. (2024) The recommendations address gaps in existing surveillance and better leverage ongoing efforts.

#### Recommendations for Surveillance

***1. Develop and implement a novel and nimble surveillance system to monitor use of high-concentration forms of cannabis.**** Rationale:* Present surveillance systems are not sufficiently dynamic to track the marketplace.

With a focus on states with regulated cannabis markets, create a new surveillance system of cannabis consumers with the objective of monitoring high-concentration forms of cannabis. The population at greatest risk of harms associated with cannabis use comprises individuals who use it heavily, regularly, and for the longest duration. Although these individuals are represented in population-based surveys, such as the NSDUH and BRFSS, those surveillance systems lack needed depth about the patterns of use and their consequences (Table 2). Additionally, these ongoing surveys cannot quickly and flexibly respond to new products in the marketplace and how they influence use patterns.

There are examples of similar opportunistic surveillance systems that use purposeful sampling, rather than representative sampling, to identify the population of interest. For example, the NIH (2024) and National Institute on Drug Abuse (NIDA) (2024) funded the implementation of the National Drug Early Warning System (NDEWS) (2024), which included 16 areas around the US. One primary goal was to track and identify emerging drugs and drug use patterns. The NDEWS conducted intercept surveys, recruiting people in public settings to complete a brief survey about illicit drug use.

Building on this model, workshop participants proposed leveraging dispensaries and the point-of-sale to recruit customers to participate in a surveillance approach that would rapidly generate data on customers and dispensaries. This strategy deploys rapid and nimble research methods that can respond to emerging issues as products in the marketplace change and questions are raised concerning risks. Specifically, this could be an opportunity to recruit individuals into qualitative, mixed methods, and/or longitudinal cohort studies to answer critical questions about high-concentration cannabis use. Recruiting from a setting where nearly everyone is a member of the population of interest augments the efficiency of surveillance.

***2. Add questions to existing surveillance systems with the objective of monitoring high-concentration cannabis and adverse outcomes.**** Rationale*: Enriching current surveillance systems with additional questions would provide needed information on use of high concentration products.

The added questions should have a specific focus on high-concentration cannabis products and adverse outcomes most relevant for the population. As noted in the Epidemiological Research section, these items should be developed in concert with the development of the core exposure and outcome measure sets.

Attendees at the workshop discussed evidence for adverse effects from high-concentration products and how the risk of each outcome varies based on the population in question. For example, cannabis hyperemesis can occur with heavy frequent use, and therefore is most likely to be an adverse effect to individuals who use it regularly, but not recent or sporadic users. In the pregnant and breastfeeding population, the risk of harm of greatest concern is to the baby, such as reduction of birth weight. Thus, additions or changes to each surveillance system should carefully consider the population reached by that system and tailor questions to the most common patterns of use and likely negative outcomes.

There are a core set of domains that should be included in each population-based survey or other surveillance system (Supplemental Table 2). Tracking systems should be assessed for how well these domains are covered, and additional items should be added to ensure the items on cannabis address use of high-concentration cannabis.

***3. Elevate the coordination, synthesis, and dissemination of findings in existing data sources that could signal adverse outcomes from high-concentration cannabis**** Rationale:* Given the evolution of the marketplace, there is urgency in identifying and communicating any adverse effects of high-concentration products.

There are numerous sources of surveillance and administrative data that could be leveraged to monitor public health effects of cannabis use (e.g., emergency department data, poison control, fatalities, motor vehicle, and population-surveys). However, there are barriers and data silos that limit access to and the informativeness of these potentially useful data resources, including considerable variability across states in these data systems. States could more consistently compile relevant data to triangulate the consequences of cannabis use, particularly the emerging concern of high-concentration cannabis use.

#### Implementation of Recommendations for Surveillance

Implementation of these recommendations requires collaboration amongst the various entities and funding for the proposed advancements. The proposed new surveillance system (Recommendation 1) would come with the costs of developing and testing the proposed approach. Once developed, there would be costs for implementing and maintaining this novel system. Considering the necessity of having a timelier and more detailed grasp on use of cannabis products, costs for such a system are justified. State regulators should be able to facilitate cooperation from dispensaries to permit participant recruitment on the premise of monitoring public health issues. Adding questions to existing surveys (Recommendation 2) may come with minimal costs. The proposed questions need to be vetted through review processes, which are typically ongoing given the continued need for quality improvement. The costs are typically opportunity-costs of questions that may be removed to make space for these additions. Considering Recommendation 3, there is already ongoing administrative and surveillance data collected in states with regulated markets that can be further leveraged to monitor trends in cannabis use and harms. Triangulating multiple sources of surveillance data can provide a more complete picture of outcomes associated with high-concentration cannabis use.

## Conclusion

There are significant gaps in research on cannabis, particularly on the high-concentration THC products in the current marketplace. The limited evidence available varies significantly in methods and outcome measurements, making interpretation difficult and limiting generalizability. Assuming the marketplace continues to evolve, Fig. 1 shows how surveillance and standardizing research will support policy development, implementation, and evaluation. To implement these recommendations, multi-sectoral collaboration and coordination are needed and investment is needed for enhanced surveillance, research, and policy development. The framework (Fig. 1) shows how broadly based research meshes with policy development, implementation, and evaluation, and highlights the necessity of funding research for public health protection. The workshop did not address details of funding, including what entities should be responsible, although the states could assume leadership to support research. Academia will carry out the needed research if funding is available. Typically, academic researchers will collaborate and coordinate research activities and are likely to embrace the proposal for improved methods.

## Supplementary Information

Supplementary Material 1.

## Data Availability

No datasets were generated or analysed during the current study.

## Abbreviation

THC: Delta-9-tetrahydrocannbinol
COMET: Core Outcome Measures in Effectiveness Trials
CONSORT: Consolidated Standards of Reporting Trials
iCannToolkit: International Cannabis Toolkit
DFAQ-CU: Daily Sessions, Frequency, Age of Onset, and Quantity of Cannabis Use Inventory
NIH: National Institutes of Health
US: United States
BRFSS: Behavioral Risk Factor Surveillance System
HKCS: Healthy Kids Colorado Survey
YRBSS: Youth Risk Behavior Surveillance System
DUI: Driving Under the Influence
NSDUH: National Survey on Drug Use and Health
ICPS: International Cannabis Policy Study
PRAMS: Pregnancy Risk Assessment Monitoring System
FDA: Food and Drug Administration
NIDA: National Institute on Drug Abuse
MTF: Monitoring the Future
NPDS: National Poison Data System
WYAHS: Washington Young Adult Health Survey

## References

[CR1] American College of Medical Toxicology. Toxicology Investigators Consortium (ToxIC) Homepage Phoenix, AZ. 2024 [Available from: https://www.acmt.net/core-registry/. Accessed 10 May 2025.

[CR2] Berl V, Hurd YL, Lipshutz BH, Roggen M, Mathur EJ, Evans M. A Randomized, Triple-Blind, Comparator-Controlled Parallel Study Investigating the Pharmacokinetics of Cannabidiol and Tetrahydrocannabinol in a Novel Delivery System, Solutech, in Association with Cannabis Use History. Cannabis Cannabinoid Res. 2022;7(6):777–89. 35787693 10.1089/can.2021.0176 PMC9784610

[CR3] Bero L, Lawrence R, Oberste JP, Li T, Leslie L, Rittiphairoj T, Piper C, Wang GS, Brooks-Russell A, Yim TW, Tung G, Samet JM. Health Effects of High-Concentration Cannabis Products: Scoping Review and Evidence Map. Am J Public Health. 2023;113(12):1332–42. 37939329 10.2105/AJPH.2023.307414 PMC10632847

[CR4] Bialas P, Fitzcharles MA, Klose P, Hauser W. Long-term observational studies with cannabis-based medicines for chronic non-cancer pain: A systematic review and meta-analysis of effectiveness and safety. Eur J Pain. 2022;26(6):1221–33. 35467781 10.1002/ejp.1957

[CR5] Cannabis Research and Policy Project Team. A Scoping Review on Health Effects of High-Concentration Cannabis Products: Findings on Key Policy Questions. Colorado School of Public Health; 2023. Contract No.: 76.

[CR6] Cannabis Control Commission Common Wealth of Massachusetts. Homepage. 2024 [Available from: https://www.masscannabiscontrol.com/. Accessed 10 May 2025.

[CR7] Cash MC, Cunnane K, Fan C, Romero-Sandoval EA. Mapping cannabis potency in medical and recreational programs in the United States. PLoS ONE. 2020;15(3): e0230167. 32214334 10.1371/journal.pone.0230167 PMC7098613

[CR8] Centers for Disease Control and Prevention (CDC), Adolescent and School Health. School Health Profiles. 2022 [Available from: https://www.cdc.gov/healthyyouth/data/profiles/index.htm. Accessed 10 May 2025.

[CR9] Centers for Disease Control and Prevention. Behavioral Risk Factor Surveillance System (BRFSS). 2022 [updated May 17, 2024. Available from: https://www.cdc.gov/brfss/index.html. Accessed 10 May 2025.

[CR10] Centers for Disease Control and Prevention (CDC). Pregnancy Risk Assessment Monitoring System (PRAMS). 2024 [Available from: https://www.cdc.gov/prams/index.html. Accessed 10 May 2025.

[CR11] Centers for Disease Control and Prevention. Youth Risk Behavior Surveillance System (YRBSS). 2021 [Available from: https://www.cdc.gov/yrbs/index.html. Accessed 10 May 2025.

[CR12] Chapekis A, Shah S. Most Americans now live in a legal marijuana state – and most have at least one dispensary in their county Washington, D.C.: Pew Research Center; 2024 [updated February 29, 2024. Available from: https://www.pewresearch.org/short-reads/2024/02/29/most-americans-now-live-in-a-legal-marijuana-state-and-most-have-at-least-one-dispensary-in-their-county/. Accessed 10 May 2025.

[CR13] Colorado Department of Public Health & Environment (CDPHE). Baby and You Survey. 2024 [Available from: https://cdphe.colorado.gov/BabyandYou. Accessed 10 May 2025.

[CR14] Colorado Department of Revenue, Marijuana Enforcement Division. Regulated Marijuana Market Update. 2020. Accessed 10 May 2025.

[CR15] Colorado Department of Public Health & Environment (CDPHE). Health eMoms Survey Data; 2024 [Available from: https://cdphe.colorado.gov/center-for-health-and-environmental-data/survey-research/health-emoms/health-emoms-survey-data.] Accessed 10 May 2025.

[CR16] Colorado Department of Public Health & Environment (CDPHE). Healthy Kids Colorado Survey (HKCS) Homepage. 2024 [Available from: https://cdphe.colorado.gov/hkcs. Accessed 10 May 2025.

[CR17] Committee on the Public Health Consequences of Changes in the Cannabis Policy Landscape (Washington District of Columbia). Cannabis Policy Impacts Public Health and Health Equity. Washington: National Academies Press. 2024. 39602559

[CR18] Connors N, Kosnett MJ, Kulig K, Nelson LS, Stolbach AI. ACMT Position Statement: Interpretation of Urine for Tetrahydrocannabinol Metabolites. J Med Toxicol. 2020;16(2):240–2. 31939053 10.1007/s13181-019-00753-8 PMC7099115

[CR19] Cuttler C, Spradlin A. Measuring cannabis consumption: Psychometric properties of the Daily Sessions, Frequency, Age of Onset, and Quantity of Cannabis Use Inventory (DFAQ-CU). PLoS ONE. 2017;12(5): e0178194. 28552942 10.1371/journal.pone.0178194 PMC5446174

[CR20] DeGregorio MW, Wurz GT, Montoya E, Kao CJ. A comprehensive breath test that confirms recent use of inhaled cannabis within the impairment window. Sci Rep. 2021;11(1):22776. 34815467 10.1038/s41598-021-02137-x PMC8611040

[CR21] ElSohly MA, Feng S. delta 9-THC metabolites in meconium: identification of 11-OH-delta 9-THC, 8 beta,11-diOH-delta 9-THC, and 11-nor-delta 9-THC-9-COOH as major metabolites of delta 9-THC. J Anal Toxicol. 1998;22(4):329–35. 9681337 10.1093/jat/22.4.329

[CR22] Fabritius M, Favrat B, Chtioui H, Battistella G, Annoni JM, Appenzeller M, et al. THCCOOH concentrations in whole blood: are they useful in discriminating occasional from heavy smokers? Drug Test Anal. 2014;6(1–2):155–63. 24173827 10.1002/dta.1581

[CR23] Fadus MC, Valadez EA, Bryant BE, Garcia AM, Neelon B, Tomko RL, et al. Racial Disparities in Elementary School Disciplinary Actions: Findings From the ABCD Study. J Am Acad Child Adolesc Psychiatry. 2021;60(8):998–1009. 33359407 10.1016/j.jaac.2020.11.017 PMC8860403

[CR24] Freeman AM, Petrilli K, Lees R, Hindocha C, Mokrysz C, Curran HV, et al. How does cannabidiol (CBD) influence the acute effects of delta-9-tetrahydrocannabinol (THC) in humans? A Systematic Review Neurosci Biobehav Rev. 2019;107:696–712. 31580839 10.1016/j.neubiorev.2019.09.036

[CR25] Gette JA, Littlefield AK, Victor SE, Schmidt AT, Garos S. Evaluation of the Daily Sessions, Frequency, Age of Onset, and Quantity of Cannabis Use Questionnaire and its Relations to Cannabis-Related Problems. Cannabis. 2023;6(3):64–86. 38035173 10.26828/cannabis/2023/000161 PMC10683753

[CR26] Hammond D. International Cannabis Policy Study Homepage Waterloo, Ontario, Canada: University of Waterloo. 2024 [Available from: https://cannabisproject.ca/.

[CR27] Hammond D. International Cannabis Policy Study (ICPS) Waterloo, Canada2024 [Available from: https://davidhammond.ca/projects/drugs-policy/illicit-drug-use-among-youth/.

[CR28] Harlow AF, Leventhal AM, Barrington-Trimis JL. Closing the Loophole on Hemp-Derived Cannabis Products: A Public Health Priority. JAMA. 2022;328(20):2007–8. 36331491 10.1001/jama.2022.20620 PMC10406389

[CR29] Huestis MA. Human cannabinoid pharmacokinetics. Chem Biodivers. 2007;4(8):1770–804. 17712819 10.1002/cbdv.200790152 PMC2689518

[CR30] Henthorn TK, Wang GS, Dooley G, Brooks-Russell A, Wrobel J, Limbacher S, et al. Dose Estimation Utility in a Population Pharmacokinetic Analysis of Inhaled Δ9-Tetrahydrocannabinol Cannabis Market Products in Occasional and Daily Users. Ther Drug Monit. 2024. 10.1097/FTD.0000000000001224 PMC11389879 39235358

[CR31] Hryhorowicz S, Walczak M, Zakerska-Banaszak O, Slomski R, Skrzypczak-Zielinska M. Pharmacogenetics of Cannabinoids. Eur J Drug Metab Pharmacokinet. 2018;43(1):1–12. 28534260 10.1007/s13318-017-0416-z PMC5794848

[CR32] Hubbard JA, Hoffman MA, Ellis SE, Sobolesky PM, Smith BE, Suhandynata RT, et al. Biomarkers of Recent Cannabis Use in Blood, Oral Fluid and Breath. J Anal Toxicol. 2021;45(8):820–8. 34185831 10.1093/jat/bkab080

[CR33] Initiative C. core outcome measures in effectiveness trials; 2024. Available from: https://comet-initiative.org/. Accessed 10 May 2025.

[CR34] Jensen TL, Wu F, McMillin GA. Detection of in utero Exposure to Cannabis in Paired Umbilical Cord Tissue and Meconium by Liquid Chromatography-Tandem Mass Spectrometry. Clin Mass Spectrom; 2019.14 Pt B:115–23. 10.1016/j.clinms.2019.01.002 PMC8669442 34917768

[CR35] Kandi V, Vadakedath S. Clinical Trials and Clinical Research: A Comprehensive Review. Cureus. 2023;15(2): e35077. 36938261 10.7759/cureus.35077 PMC10023071

[CR36] Karschner EL, Schwilke EW, Lowe RH, Darwin WD, Pope HG, Herning R, et al. Do Delta9-tetrahydrocannabinol concentrations indicate recent use in chronic cannabis users? Addiction. 2009;104(12):2041–8. 19804462 10.1111/j.1360-0443.2009.02705.x PMC2784185

[CR37] Kim J, de Castro A, Lendoiro E, Cruz-Landeira A, López-Rivadulla M, Concheiro M. Detection of in utero cannabis exposure by umbilical cord analysis. Drug Test Anal. 2018;10(4):636–43. 28948698 10.1002/dta.2307

[CR38] Kosnett MJ, Ma M, Dooley G, Wang GS, Friedman K, Brown T, et al. Blood cannabinoid molar metabolite ratios are superior to blood THC as an indicator of recent cannabis smoking. Clin Toxicol (Phila). 2023;61(5):355–62. 37293900 10.1080/15563650.2023.2214697 PMC10481452

[CR39] Li T, Wang GS, Brooks-Russell A, Tung G, Leslie L, Rittiphairoj T, et al. Methodological Challenges and Actionable Recommendations in Studying the Health Effects of High-Concentration THC Products. In: Health CSoP, Colorado Uo, editors. 2024. 10.1093/aje/kwae421 39475086

[CR40] Li T, Wang GS, Brooks-Russell A, Tung G, Leslie L, Rittiphairoj T, et al. Methodological challenges and actionable recommendations in studying the health effects of high-concentration THC products. Am J Epidemiol. 2024. 10.1093/aje/kwae421 39475086

[CR41] Lee D, Huestis MA. Current knowledge on cannabinoids in oral fluid. Drug Test Anal. 2014;6(1–2):88–111. 23983217 10.1002/dta.1514 PMC4532432

[CR42] Lile JA, Kelly TH, Charnigo RJ, Stinchcomb AL, Hays LR. Pharmacokinetic and pharmacodynamic profile of supratherapeutic oral doses of Delta(9) -THC in cannabis users. J Clin Pharmacol. 2013;53(7):680–90. 23754596 10.1002/jcph.90 PMC3691290

[CR43] Lorenzetti V, Hindocha C, Petrilli K, Griffiths P, Brown J, Castillo-Carniglia A, et al. The International Cannabis Toolkit (iCannToolkit): a multidisciplinary expert consensus on minimum standards for measuring cannabis use. Addiction. 2022a;117(6):1510–7. 34590359 10.1111/add.15702 PMC9298052

[CR44] Lorenzetti V, Hindocha C, Petrilli K, Griffiths P, Brown J, Castillo-Carniglia A, et al. The iCannToolkit: a tool to embrace measurement of medicinal and non-medicinal cannabis use across licit, illicit and cross-cultural settings. Addiction. 2022b;117(6):1523–5. 35289010 10.1111/add.15855 PMC9314823

[CR45] Lucas CJ, Galettis P, Schneider J. The pharmacokinetics and the pharmacodynamics of cannabinoids. Br J Clin Pharmacol. 2018;84(11):2477–82. 30001569 10.1111/bcp.13710 PMC6177698

[CR46] MacCallum CA, Lo LA, Pistawka CA, Christiansen A, Boivin M. Cannabis vaporisation: Understanding products, devices and risks. Drug Alcohol Rev. 2024;43(3):732–45. 38124429 10.1111/dar.13800

[CR47] MacCallum CA, Lo LA, Pistawka CA, Boivin M. A Clinical Framework for Evaluating Cannabis Product Quality and Safety. Cannabis Cannabinoid Res. 2023;8(3):567–74. 35049330 10.1089/can.2021.0137 PMC10249738

[CR48] Miech RA, Patrick ME. Monitoring the Future Homepage: University of Michigan; 2024 [Available from: https://monitoringthefuture.org/.

[CR49] Metz TD, McMillin GA, Silver RM, Allshouse AA, Heard K, Jensen TL, et al. Quantification of prenatal marijuana use: evaluation of the correlation between self-report, serum, urine and umbilical cord assays among women delivering at two urban Colorado hospitals. Addiction. 2022;117(1):172–81. 34142398 10.1111/add.15606 PMC8664979

[CR50] Nadulski T, Sporkert F, Schnelle M, Stadelmann AM, Roser P, Schefter T, et al. Simultaneous and sensitive analysis of THC, 11-OH-THC, THC-COOH, CBD, and CBN by GC-MS in plasma after oral application of small doses of THC and cannabis extract. J Anal Toxicol. 2005;29(8):782–9. 16356335 10.1093/jat/29.8.782

[CR51] National Capital Poison Center. Poison Control. 2024 [Available from: https://poisoncenters.org/national-poison-data-system. Accessed 10 May 2025.

[CR52] National Drug Early Warning System. Homepage. 2024 [updated July 8, 2024. Available from: https://ndews.org. Accessed 10 May 2025.

[CR53] National Institutes of Health. Homepage. 2024 [Available from: https://www.nih.gov/.] Accessed 10 May 2025.

[CR54] National Highway Traffic Safety Administration (NHTSA), United States Department of Transportation. Fatality Analysis Reporting System (FARS). 2024 [Available from: https://www.nhtsa.gov/research-data/fatality-analysis-reporting-system-fars. Accessed 10 May 2025.

[CR55] NIH National Institute on Drug Abuse. Homepage. 2024 [Available from: https://nida.nih.gov/.] Accessed 10 May 2025.

[CR56] NIH Pragmatic Trials Collaboratory. Updated Template Provides Guidance for Reporting of Pragmatic Trial Results. 2024 [Available from: https://rethinkingclinicaltrials.org/news/february-22-2024-updated-template-provides-guidance-for-reporting-of-pragmatic-trial-results/. Accessed 10 May 2025.

[CR57] Newmeyer MN, Swortwood MJ, Barnes AJ, Abulseoud OA, Scheidweiler KB, Huestis MA. Free and Glucuronide Whole Blood Cannabinoids’ Pharmacokinetics after Controlled Smoked, Vaporized, and Oral Cannabis Administration in Frequent and Occasional Cannabis Users: Identification of Recent Cannabis Intake. Clin Chem. 2016;62(12):1579–92. 27899456 10.1373/clinchem.2016.263475

[CR58] Pacula RL, Pessar SC, Zhu J, Smart R. Federal Regulations of Cannabis for Public Health in the United States Los Angeles, CA: University of Southern California. 2022 [updated July 18, 2022. Available from: https://healthpolicy.usc.edu/research/federal-regulations-of-cannabis-for-public-health-in-the-u-s/.

[CR59] Pandya V, Wilker C, McMillin GA. Can Umbilical Cord and Meconium Results Be Directly Compared? Analytical Approach Matters J Anal Toxicol. 2023;47(1):96–105. 35707888 10.1093/jat/bkac037 PMC9942436

[CR60] Public Health Institute. Guiding Cannabis Policies that Protect Youth and Health. 2020 [Available from: https://www.phi.org/about/impacts/guiding-cannabis-policies-that-protect-youth-health/. Accessed 10 May 2025.

[CR61] Robertson MB, Li A, Yuan Y, Jiang A, Gjerde H, Staples JA, et al. Correlation between oral fluid and blood THC concentration: A systematic review and discussion of policy implications. Accid Anal Prev. 2022;173: 106694. 35640367 10.1016/j.aap.2022.106694

[CR62] Rozanc J, Klumpers LE, Huestis MA, Tagen M. Tolerability of High-Dose Oral Δ9-THC: Implications for Human Laboratory Study Design. Cannabis and Cannabinoid Research. 2024;9(2):437–48. 38377580 10.1089/can.2023.0209

[CR63] Schwilke EW, Karschner EL, Lowe RH, Gordon AM, Cadet JL, Herning RI, et al. Intra- and intersubject whole blood/plasma cannabinoid ratios determined by 2-dimensional, electron impact GC-MS with cryofocusing. Clin Chem. 2009;55(6):1188–95. 19264857 10.1373/clinchem.2008.114405 PMC3197018

[CR64] Solmi M, De Toffol M, Kim JY, Choi MJ, Stubbs B, Thompson T, et al. Balancing risks and benefits of cannabis use: umbrella review of meta-analyses of randomised controlled trials and observational studies. BMJ. 2023;382: e072348. 37648266 10.1136/bmj-2022-072348 PMC10466434

[CR65] Substance Abuse and Mental Health Services Administration (SAMHSA). National Survey on Drug Use and Health (NSDUH); 2024 [Available from: https://www.samhsa.gov/data/data-we-collect/nsduh-national-survey-drug-use-and-health. Accessed 10 May 2025.

[CR66] United States Food and Drug Administration (FDA). FDA Adverse Event Reporting System (FAERS) Public Dashboard. 2023 [updated December 7, 2023. Available from: https://www.fda.gov/drugs/questions-and-answers-fdas-adverse-event-reporting-system-faers/fda-adverse-event-reporting-system-faers-public-dashboard. Accessed 10 May 2025.

[CR67] Vandrey R, Herrmann ES, Mitchell JM, Bigelow GE, Flegel R, LoDico C, et al. Pharmacokinetic Profile of Oral Cannabis in Humans: Blood and Oral Fluid Disposition and Relation to Pharmacodynamic Outcomes. J Anal Toxicol. 2017;41(2):83–99. 28158482 10.1093/jat/bkx012 PMC5890870

[CR68] Ward SJ, Lichtman AH, Piomelli D, Parker LA. Cannabinoids and Cancer Chemotherapy-Associated Adverse Effects. J Natl Cancer Inst Monogr. 2021;2021(58):78–85. 34850893 10.1093/jncimonographs/lgab007 PMC8848502

[CR69] Washington Young Adult Health Survey. Homepage. 2014 [Available from: https://sites.uw.edu/uwwyahs/.] Accessed 10 May 2025.

