# Supplementary material for: Enhancing Methods for Research on Cannabis: A Workshop Report
Source: J Cannabis Res. 2025 Sep 29;7:69. doi: 10.1186/s42238-025-00314-7 (PMC12482547; doi:10.1186/s42238-025-00314-7)
Supplement: Supplementary file 1 — Supplementary Material 1. [file 42238_2025_314_MOESM1_ESM.docx]

**Supplemental Material**

**Background**

The progressive legalization of medical and recreational cannabis markets at the state-level led to the rapid growth of medical and recreational cannabis markets. This growth in state markets occurred along two dimensions: the number of outlets/dispensaries (i.e., suppliers) and the type of products sold in stores. Product diversification and innovation resulted in a decline in the share of the total market represented by flower, due to an increasing share of the market being occupied by vape pens, edibles, concentrates, and pre-rolled joints that can have additional delta-9-tetrahydrocannabinol (THC) infused on the bud they contain. The concentration of THC in these new products is far higher than even a decade ago (Table 4 of Manuscript). Greater availability of cannabis products and higher THC concentrations magnify the effects of cannabis use, including the risks for harmful effects. Consequently, there is an imperative for states to more effectively regulate cannabis markets to address the potential adverse effects on public health and to provide information to the public on products in the marketplace.

The Colorado General Assembly, concerned by the availability of high-concentration THC products in the state’s cannabis marketplace, passed House Bill 21-1317 (HB 1317) in 2021. Among its provisions, HB 1317 called on the Colorado School of Public Health to “…conduct a systematic review of all available scientific evidence-based research regarding the possible physical and mental health effects of high-potency THC marijuana and marijuana concentrates regardless of the location of the research.” With this direction, the Cannabis Research and Policy Team, constituted to address this charge, completed a scoping review of the literature. The review identified 452 studies through June 30, 2022, that met the criteria for relevance to the critical general policy question: What are the public health consequences of the increasing availability of these newer high-concentration THC products? The scoping review (1) included human studies of any epidemiological and clinical design, without restrictions based on age, sex, health status, country, or outcome measured, as long as THC concentrations were reported and met the criterion for inclusion, or a known high-concentration THC product was investigated.

Overall, the evidence foundation was considered weak for addressing this critical question and supporting evidence-informed decision-making. The flaws included limitations posed by the study designs and methods, and by relevance to the current marketplace. However, there is evidence about use of cannabis and THC generally that is highly relevant to policy formulation, e.g., protecting the fetus and the infant from THC exposure and limiting use of cannabis products in adolescents and young adults whose brains are still developing. Considering the research specifically on the newer and higher concentration products, the quality of the evidence was limited by highly variable and incomplete approaches to measuring cannabis use and THC exposure. Common problems included not fully covering the dimensions of use, e.g., frequency and duration, nor capturing the specific products used. Consequently, the literature available does not meet the evidence needs for decision-making at present, particularly given the mismatch between the research findings published and THC concentrations of cannabis products now in use. There are also populations at increased risk, e.g., the fetus and the infant, for whom focused research is needed. These limitations of the evidence foundation for decision-making on policies related to high-concentration cannabis products need to be addressed with research relevant to today’s marketplace and patterns of use. These findings from the scoping review are now reported in a commentary in the *American Journal of Public Health* that calls for the enhancement of methods for research on cannabis.(2)

**Workshop Framework**

Workshop participants adopted a general framework for capturing how research and surveillance data influence policy and the marketplace (Figure 1 of Manuscript). Public health surveillance data, describing the marketplace, use patterns, and sentinel health events, are placed centrally as such data can identify warnings for issues that require follow-up. Currently, surveys at the international, national, and state levels track various aspects of cannabis use (Table 2 of Manuscript). Tracking the products in the marketplace is also a critical aspect of surveillance, as research typically lags behind the products under investigation versus those available in the marketplace. Together, these data sources will help identify issues concerning the public’s health and where further research is needed, as illustrated in the middle ring of research activities surrounding “surveillance” at the core. The outermost ring is the policy cycle, a loop that includes policy development, policy implementation, marketplace innovation partially in response to those policies, and policy evaluation. The figure shows the foundational role that research plays, whether basic, clinical, epidemiological, or policy-directed, in informing our knowledge of the relationship between policy, individual behavior, and health outcomes. The “cycle” recognizes the dynamic interactions that exist between policy, the cannabis marketplace, and surveillance, and how different types of research are needed to truly understand these dynamics. For example, policies that reduce the availability of particular types of products (e.g., high-concentration THC products) will impact the legal marketplace (e.g., removing them from stores) but may or may not impact health outcomes (e.g., car accidents) if consumers choose to obtain those restricted products from an illegal source rather than choosing a legal low-concentration THC product. Surveillance of adverse events associated with consumption of high-concentration THC products can indicate that there may be a positive or negative effect of such a policy, but careful research is needed before we can definitively know that the policy itself generated the change linked to the adverse events, or if the change was driven by an unaccounted factor (e.g., people drive less because of ride service applications becoming available in the same jurisdiction). Workshop participants also discussed terminology for cannabis use related to determinants of THC exposure dose reaching target receptors, particularly in the brain (Figure 2 of Manuscript). The figure captures key factors in the pathway from cannabis products to health effects, whether beneficial or adverse. It portrays the various dimensions of cannabis use that are potentially relevant to health outcomes. The first step that participants considered was terminology related to cannabis use (e.g., THC product concentration and amount, exposure, and dose parameters). These terms were applied variably and participants deemed a consensus on terminology that would be useful. The report of the group addressing clinical research provides recommendations on terminology. Reports and recommendations from each group follow.

**Colorado Based Study**

As one example of compiling relevant data, Colorado provides established an oversight committee and funded health department staff to periodically examine and compile all relevant surveillance data associated with cannabis use into a biennial report provided to the state legislature(3), and available to the public. These data sources include population-based surveys, such as the BRFSS, PRAMS, and the Colorado-specific surveillance systems(4, 5). It also includes poison center call data and hospitalization data. However, even in a state with an established regulated market, there are more data sources of data that could be leveraged. For example, the drug testing associated with motor vehicle crashes and DUIs, data associated with crimes and delinquency (e.g., school disciplinary and absenteeism data, and retail market data (e.g., sales, taxes, price per unit, licensing, testing data, and other data) could be further integrated with public health data systems.

**Supplemental Table 1:** Participants of the Methods Workshop.

| **Attendees** | **Affiliation** |
| --- | --- |
| Jessica Barrington-Trimis, PhD  *(Epidemiological Studies)* | University of Southern California |
| Lisa Bero, PhD*  *(Epidemiological Studies)* | Colorado School of Public Health |
| Ashley Brooks-Russell, PhD, MPH*  *(Surveillance)* | Colorado School of Public Health |
| Meghan Buran, MPH* | Colorado School of Public Health |
| Julia Dilley, PhD, M.E.S.  *(Surveillance)* | Oregon Health Authority |
| Darin Erickson, PhD  *(Epidemiological Studies)* | University of Minnesota |
| Marilyn Huestis, PhD  *(Clinical Research)* | Thomas Jefferson University |
| Kent Hutchison, PhD**  *(Clinical Research)* | University of Colorado School of Medicine |
| Thomas L. Jeanne, MD, MPH  *(Epidemiological Studies)* | Oregon Health Authority |
| Heather Kimmel, PhD  *(Policy Research)* | National Institute on Drug Abuse & National Institutes of Health |
| Michael Kosnett, MD, MPH  *(Clinical Research)* | Colorado School of Public Health |
| David J. Kroll, PhD  *(Clinical Research)* | University of Colorado School of Pharmacy |
| Stephen Lankenau, PhD  *(Surveillance)* | Drexel University |
| Richard Miech, PhD  *(Surveillance)* | University of Michigan |
| Rosalie Liccardo Pacula, PhD  *(Policy Research)* | University of Southern California |
| Paula Riggs, MD**  *(Epidemiological Studies)* | University of Colorado School of Medicine |
| Jonathan M. Samet, MD, MS* | Colorado School of Public Health |
| Neeloofar Soleimanpour, MPH* | Colorado School of Public Health |
| Steven Teutsch, MD, MPH  *(Policy Research)* | University of Southern California |
| Gregory Tung, PhD, MPH*  *(Policy Research)* | Colorado School of Public Health |
| George Sam Wang, MD*  *(Clinical Research)* | University of Colorado School of Medicine |
| * Indicates attendees who are members of the Colorado School of Public Health Cannabis Research & Policy Project Team.  ** Indicates attendees who are members of the House Bill 21-1317 Scientific Review Council. | |

**Supplemental Table 2:** Core set of domains that should ideally be included in each population-based survey or other surveillance system.

| All forms/products of cannabis used (e.g., flower, concentrates, edibles) | An example is the International Cannabis Policy Survey:  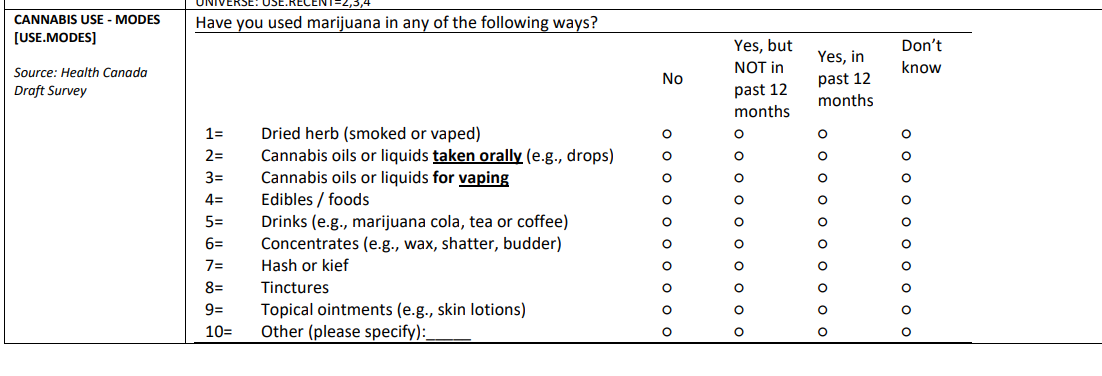 |
| --- | --- |
| The typical form/product used  For example, the Healthy Kids Colorado Survey asks about all products used (as in the item above), followed by the most common mode used: | 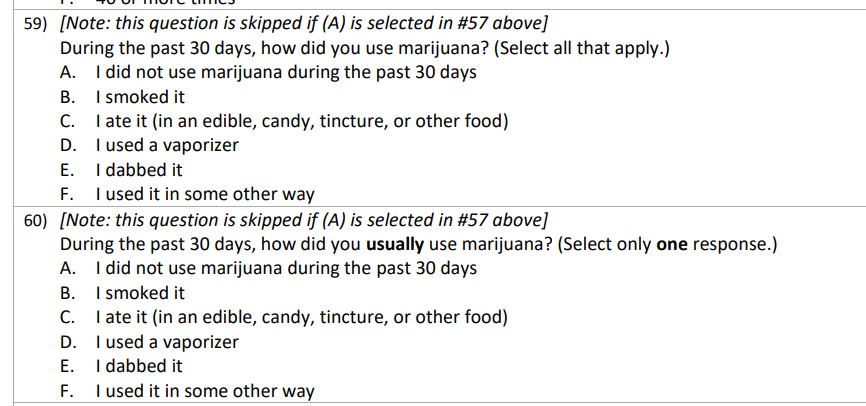 |
| Current use (past 30 days) | This is typically already included on most surveillance systems, as the most minimal of items. |
| Duration (initiation of use/onset) | For adolescent systems, this item typically asks about the age of first use. For adults, this may be years of regular use or age of regular use. |
| Hits/dose (specific to mode) - Intensity or frequency of use, as appropriate to the population and product/mode | Some assessment of the intensity or frequency of use should be asked for adolescents, this may be the number of days or times used in the past month. For adults with regular use, this could be the number of times used per day, or the amount used in a day, week, or month. For those with a regular pattern of use, asking about the amount spent or weight used may also provide insight.  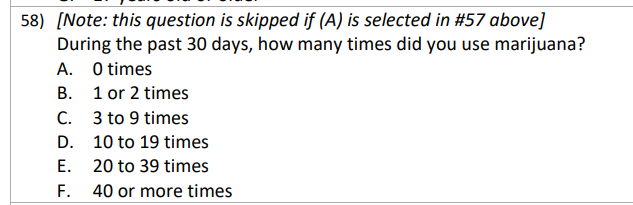 |
| Reason for use: Proportion used for “recreational” use vs. medical (including self-medication) | Asking about reasons for use is particularly helpful for pregnant populations and for adult populations to understand the balance between medical and recreational cannabis use among adults. |
| Intentional breaks: Have you stopped for a week or more for any reason | - - Have you tried to quit unsuccessfully?   - Contemplating changes in use   Questions about cutting back or quitting, whether success of not, can indicate overuse. |
| Risky behaviors: used before/during any of the following: driving, work, school | Asking about risky behaviors following acute use can indicate problematic use. For adolescents, examples of particularly problematic use would include use during the school day, or while driving. |
| Used too much such that you felt you had overused (e.g., paranoia, black-out, “overdose”) | Many surveillance systems ask about some characteristics of use, as a minimum set of items. However, it is uncommon for there to be items about consequences of misuse or over-use, which has utility for tracking consequences of the use of concentrates. |
| Source: Where did you get it? (friend, dispensary, black market) | Particularly for populations under 21, asking about how cannabis is obtained can provide insight to the size and strength of the illegal market. |
| [optional] Perceived risk and cultural acceptance | When there is time to ask additional questions, it may be useful to add items that assess the attitudes and perceptions associated with cannabis use. This may include the perceived risk or harmfulness of use. For adolescents, asking about perceived availability, perception of peer use, and perceptions of consequences provides a barometer of overall acceptance of use, which has been shown to be associated with likelihood of later use. |

**References:**

1. Cannabis Research and Policy Project Team. A Scoping Review on Health Effects of High-Concentration Cannabis Products: Findings on Key Policy Questions. Colorado School of Public Health; 2023. Contract No.: 76.

2. Li T, Wang GS, Bero L, Brooks-Russell A, Tung G, Samet JM. Enhancing Methodological Approaches for Studying Health Effects of High-Concentration THC Products. American Journal of Public Health. 2024(114):S639-S44.

3. Colorado Department of Public Health & Environment (CDPHE). Monitoring Health Concerns Related to Marijuana in Colorado: 2022 Summary. 2023 January 31, 2023.

4. Colorado Department of Public Health & Environment (CDPHE). Baby and You Survey 2024 [Available from: <https://cdphe.colorado.gov/BabyandYou>.

5. Colorado Department of Public Health & Environment (CDPHE). Health eMoms Survey Data 2024 [Available from: <https://cdphe.colorado.gov/center-for-health-and-environmental-data/survey-research/health-emoms/health-emoms-survey-data>.
